# Molecular insights into CRIP1 as an immunometabolic regulator revealed by CRIP1 knockout and single-cell transcriptomics

**DOI:** 10.3389/fimmu.2026.1762474

**Published:** 2026-03-26

**Authors:** DoYeon Kim, Bok Hee Woo, Seon Hyun Kim, Hye Jung Kim, JiHye Lee, Hae Ryoun Park, Jae-Min Song

**Affiliations:** 1 Department of Oral Pathology, School of Dentistry, Pusan National University, Yangsan, Republic of Korea; 2 Periodontal Disease Signaling Network Research Center, Dental and Life Science Institute, School of Dentistry, Pusan National University, Yangsan, Republic of Korea; 3 Department of Oral and Maxillofacial Surgery, Dental and Life Science Institute, School of Dentistry, Pusan National University, Yangsan, Republic of Korea; 4 Department of Oral and Maxillofacial Surgery, Dental Research Institute, Pusan National University Dental Hospital, Yangsan, Republic of Korea

**Keywords:** CRIP1, iron metabolism, NAFLD, oxidative phosphorylation (OXPHOS), periodontitis, zinc metabolism

## Abstract

Cysteine-rich intestinal protein 1 (CRIP1) was first identified as a metabolism-related gene nearly four decades ago, yet its precise biological function remains poorly defined. More recently, CRIP1 has been implicated in several cancers, including hepatocellular carcinoma, acute myeloid leukemia, multiple myeloma, and melanoma, where it can act as either a pro-tumorigenic factor or a tumor suppressor. In contrast, its physiological roles under non-malignant conditions have been only minimally explored, and current understanding remains largely confined to early speculative links with zinc transport and metal ion metabolism. This is the first to comprehensively dissect the basic functions of CRIP1, addressing both immune regulation and metabolism. We first sought to define CRIP1’s involvement in hepatic metabolism by analyzing liver tissues from CRIP1 knockout (KO) and wild-type (WT) mice under basal conditions, focusing on zinc transport, iron metabolism, and mitochondrial oxidative phosphorylation identified through transcriptomic analysis. In parallel, we evaluated how CRIP1 deficiency modulates metabolic and immune responses under chronic low-grade inflammation induced by oral gavage with periodontal pathogens, assessing CRIP1-dependent changes. Furthermore, we investigated the relationship between CRIP1 expression and inflammatory gene profiles using single cell RNA sequencing data of peripheral mononuclear cells from healthy individuals and periodontitis patients. By comparing pro-inflammatory and anti-inflammatory gene expression according to CRIP1 expression levels across diverse immune cell subsets, we aimed to clarify whether CRIP1 is more likely to contribute to pro-inflammatory or anti-inflammatory regulation. This integrated approach provides new insights into the fundamental immunomodulatory and metabolic role of CRIP1.

## 1 Introduction

Cysteine-rich intestinal protein 1 (CRIP1) was first identified by Birkenmeier EH et al. through screening of small intestinal cDNA libraries from rats and mice nearly four decades ago. It was initially classified as a putative member of the ferredoxin superfamily based on computer-assisted comparisons, although this classification lacked molecular evidence. This suggested a potential role in electron transport and iron metabolism through iron binding (1). Subsequent work by Hempe et al. proposed that CRIP1 functions as a key molecule in intracellular zinc transport by competitively binding zinc with metallothionein during intestinal zinc absorption (2). Later, Pérez-Alvarado et al. confirmed its metal-binding properties by determining the three-dimensional structure of CRIP1 using nuclear magnetic resonance spectroscopy (3). Based on these findings, CRIP1 has been assumed to regulate cellular metabolism through its metal ion-binding capability. However, despite these initial reports, mechanistic or molecular evidence underlying CRIP1’s biologic functions remain limited.

More recently, CRIP1 has been implicated in cell death pathways and tumor biology, particularly in relation to apoptosis, autophagy, and chemoresistance. For example, silencing of CRIP1 induces cell cycle arrest and apoptosis in thyroid cancer and acute myeloid leukemia (AML) cells, supporting its anti-apoptotic and tumor-promoting effects. Tang P et al. reported that CRIP1 enhances autophagy and proteasome activity by stabilizing the proteasome coactivator PA200 in multiple myeloma, thereby contributing to drug resistance (4). In addition, Wang J demonstrated that CRIP1 suppresses carnitine metabolism in hepatocellular carcinoma cells, suggesting that CRIP1 may serve as a novel regulatory factor for energy metabolism, especially in hepatocytes (5). Of note, CRIP1 is rarely detected in normal liver tissue, but is markedly increased in response to injurious stimuli, such as carbon tetrachloride (CCL_4_) administration as well as in hepatocellular carcinoma (5–7). Moreover, CRIP1 has been implicated in immune-related processes, as its expression increases in immune cells and intestinal tissues following lipopolysaccharide (LPS) administration, indicating that CRIP1 released from immune cells may systemically influence various organs (8). Accordingly, we investigated the molecular differences in liver tissue between WT and KO mice, including mice exposed to pathogens, to clarify the effects of CRIP1 deficiency.

In the present study, we aimed to elucidate the biologic functions and molecular alterations mediated by CRIP1 by comparing wild type (WT) and CRIP1 knockout (KO) mice under both basal and pathogen-exposed conditions. We also examined the effects of mild persistent inflammation induced by oral gavage with periodontal pathogens on the liver, focusing on the signaling pathways primarily regulated by CRIP1. Through these approaches, we sought to uncover insights into the physiological and pathological roles of CRIP1, particularly in the context of metal ion metabolism, immune responses, and disease progression.

## 2 Materials and methods

### 2.1 Animal model

CRIP1 KO mice were generated using CRISPR/Cas9 system with two single-guide RNAs (sgRNAs) targeting exon deletions at exon 2, 3, and 4 (sg1: ACATTGAAAGAATGAAGTGTGG, sg2: CCCAGTCCACCAGTTTACAGGAC). To ensure genetic stability, KO mice were backcrossed for seven generations. Genotyping of each mouse was confirmed by polymerase chain reaction (PCR). Six weeks old C57BL/6 WT and CRIP1 KO mice were used for study.

For oral gavage infection, mice were pretreated with antibiotics (ampicillin and metronidazole at 0.2 g/L in autoclaved drinking water) for 2 days. Antibiotic-containing water was replaced with antibiotic-free water prior to bacterial gavage. *Porphyromonas gingivalis* (*P. gingivalis*, strain 381, Korean Collection for Type Cultures, Jeongeup, Republic of Korea) and *Fusobacterium nucleatum* (*F. nucleatum*, nucleatum subspecies, Polymorphum, KCTC) were cultured anaerobically in Gifu Anaerobic Medium (GAM) broth (Shimadzu Diagnostics, Tokyo, Japan) supplemented with Hemin and vitamin K at 37°C for 24 to 48 hours. Cultured bacteria were pelleted by centrifugation at 3,000 rpm for 10 min and suspended in phosphate-buffered saline (PBS). Bacteria concentration was standardized to an optical density at 600nm (OD600) of 2.0. P*. gingivalis* and *F. nucleatum* were mixed equally and diluted 1:1 with 2% carboxymethyl cellulose (CMC). PBS without bacteria was used as a control. One hundred μL of bacterial or control suspensions were orally gavaged once daily for two weeks. Mice were deeply anesthetized with inhaled isoflurane (2–3%), and terminal blood and organ collection was performed under anesthesia. Euthanasia was then completed by carbon dioxide inhalation at a displacement rate of 30–70% of the chamber volume per minute. This study is permitted by Pusan National University Yangsan Hospital Institutional Animal Care and Use Committee (PNUYH-IACUC, 2021-018-A1C1).

### 2.2 Peripheral blood glucose level measurement and multiplex enzyme-linked immunosorbent assay

Blood glucose level was measured from tail vein blood using a commercially available glucometer Accu-Chek^®^ Guide Me (Roche Diagnostics, Mannheim, Germany). For metabolic hormones measurement, peripheral blood of the mouse was collected from abdominal aorta. After centrifuged at 4°C, 6,000 g for 10 minutes, serum was collected and stored at −80 °C until use. The serum concentration of interleukin-6 (IL-6), Tumor necrosis factor alpha (TNFα), Insulin, Leptin, and Resistin of WT and KO with or without periodontal pathogen gavage were determined using MILLIPLEX^®^ Mouse Metabolic Hormone Expanded Panel - Metabolism Multiplex Assay (Merck KGaA, Darmstadt, Germany) according to the manufacturer’s instructions. The concentration of proteins is measured using Luminex^®^ MAGPIX^®^ instrument (Diasorin, MN, USA). Cytokine and hormones concentrations were calculated using a standard curve. All experiments were performed in duplicate.

### 2.3 Transcriptomic sequencing analysis

Total RNA was extracted from mouse liver tissue using the RNeasy Kit (QIAGEN, Hilden, Germany) and quality-checked (RIN ≥ 7, rRNA ratio ≥ 1). cDNA libraries were prepared using the TruSeq Stranded Total RNA Library Prep Gold Kit (Illumina, San Diego, CA, USA) and sequenced on the NovaSeqX platforms (Illumina) with paired-end reads. Raw reads were Trimmomatic, aligned with HISAT2, and quantified with StringTie. Differentially expressed gene (DEG) analysis was performed using DESeq2 (version 1.44.0) (9, 10). Gene set enrichment analysis (GSEA) was conducted using Gene Ontology (GO) terms and Kyoto Encyclopedia of Genes and Genomes (Kegg) pathways. To compare expression levels of categorized gene sets across groups, we computed per-sample mean z-scores following established module-scoring procedures. Expression levels were normalized using DESeq2’s variance-stabilizing transformation (VST) prior to standardization.

### 2.4 Quantitative reverse transcription-polymerase chain reaction

Complementary DNA was synthesized from 500 ng of total RNA using AccuPower^®^ RocketScript™ Master Mix (Bioneer, Daejeon, Republic of Korea). qPCR was performed with AccuPower^®^ 2X GreenStar™ qPCR Master Mix (Bioneer) on a CFX Duet Real-Time PCR System (Bio-Rad, Hercules, CA, USA). Primer sequences are listed in **Supplementary Table 1**.

### 2.5 Single cell transcriptomic analysis

The single cell RNA sequencing (scRNA seq) of healthy and periodontitis patient’s peripheral blood mononuclear cell (PBMC) were used (GSE244515) (11). The data were preprocessed using Seurat package (version 5.3.1) (12). Cells with read counts under 1000 or over 50000 as well as mitochondrial gene percentage over 15% were excluded from further analysis. In addition, we removed cells with fewer than 500 genes or more than 6000 genes, as well as those with less than 85% complexity (log-transformed number of genes detected per UMI). Doublet detection was performed within the Seurat workflow using DoubletFinder, applying an expected doublet rate of 7.5% for 10x Chromium data. DoubletFinder was run with pN = 0.25 and pK = 0.09, and cells predicted as doublets were removed prior to downstream Seurat processing. Cell type annotation was performed as the reference-based mapping using Azimuth packages (13). To access the role of CRIP1 in immune cells, each cell type was divided into CRIP1-high and CRIP1-low to none based on the expression levels of CRIP1. The threshold for CRIP1-high expression is adjusted using median + k  ×  median absolute deviation (MAD) model, and optimal k value is set as 2.0. “AddModuleScore” function from Seurat package was used to calculate the M1/M2 polarization and inflammation module scores, and gene names used in calculation are listed in **Supplementary Table 2**.

### 2.6 Statistical analysis

Data analysis was conducted using R (v4.4.1) and GraphPad Prism (v10.4.2, GraphPad Software, San Diego, CA, USA). The Mann–Whitney U test compared two independent groups, while the Kruskal–Wallis test compared multiple groups. Data are presented as mean ± standard deviation. Statistical significance was indicated as follows: *; p< 0.05, **; p< 0.01, ***; p< 0.001, and ****; p< 0.0001.

## 3 Results

### 3.1 CRIP1 deficiency is associated with a basal hepatic pro-inflammatory state

**Figure 1:**
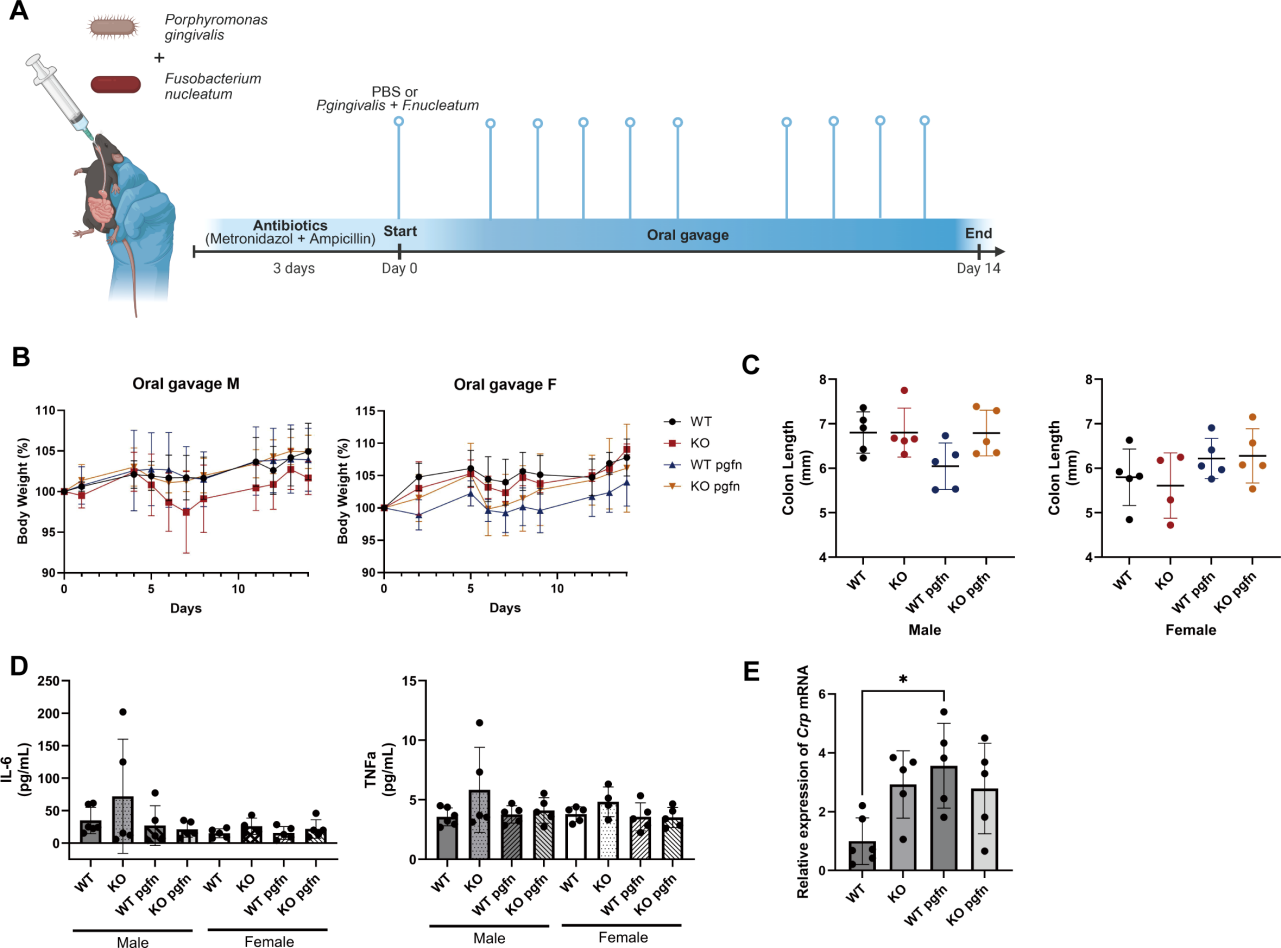
Experimental design and systemic responses to oral gavage with *Porphyromonas gingivalis* and *Fusobacterium nucleatum* in WT and KO mice. **(A)** Schematic diagram of oral gavage experiment. Male and female mice were pretreated with an antibiotic cocktail (metronidazole + ampicillin 0.2g/L each) in drinking water for 3 days, followed by repeated oral gavage with PBS (WT, KO) or a mixture of *P. gingivalis* and *F. nucleatum* (WT pgfn, KO pgfn) from day 0 to day 14. Created in BioRender. Kim, D (2026). https://BioRender.com/kst997l **(B)** Changes in body weight (percent of baseline) in male and female mice during the gavage period. Mean ± SD. **(C)** Colon length at sacrifice in male and female mice from each experimental group. Measured by ImageJ program. **(D)** Serum concentrations of IL-6 and TNFα in male and female mice. **(E)** Relative expression of *Crp* mRNA in male mice liver. **(C-E)** Each dot represents an individual animal. Mean ± SD. Kruskal-Wallis test. *p< 0.05.

To our knowledge, this is the first study to investigate the function of CRIP1 in a CRIP1 KO mouse model. Accordingly, we first assessed phenotypic changes, including morphologic characteristics, growth, and body weight gain. No significant differences were observed in morphology or growth patterns between WT and KO mice of either sex. CRIP1 deficiency did not significantly influence body weight gain during the oral gavage period or in non-infected mice, although KO males showed a slight reduction compared to WT (**Figures 1A, B**). Administration of periodontal pathogens did not significantly affect body weight in any group. Serum levels of IL-6 and TNF-α, as well as colon length, remained unchanged across all groups, indicating neither CRIP1 deficiency nor periodontal pathogen exposure induced overt systemic inflammation (**Figures 1C, D**). Notably, oral gavage with periodontal pathogens increased hepatic *Crp* (C-reactive protein) gene expression in WT mice, an effect absent in KO mice, whereas KO mice exhibited consistently elevated baseline *Crp* levels relative to WT in the absence of pathogen exposure (**Figure 1E**). These findings suggest that CRIP1 deficiency induces a mild, localized hepatic inflammatory response at baseline, such that CRIP1-deficient mice exist in a state of chronic basal hepatic inflammation but paradoxically show an attenuated response to pathogen challenges compared to WT mice. These findings suggest that CRIP1 loss raises the homeostatic hepatic inflammatory set point while impairing inflammatory responses to pathogen challenges, potentially reflecting an exhaustion-like desensitization state. In addition, the differential responses between the liver compartment and systemic markers, which were not sufficiently induced, suggest that the liver acts as an early responder to challenges, whereas systemic inflammation requires stronger or more prolonged stimuli to manifest detectable markers. Therefore, we hypothesized that CRIP1 deficiency and periodontal pathogen exposure drives specific genetic alterations in liver tissue, particularly during the early phase of the response, in pathways related to inflammatory response, metabolic process, and fatty liver-related diseases. To elucidate the molecular mechanisms underlying these observations, we performed gene expression profiling in liver tissue.

### 3.2 CRIP1 deficiency reshapes hepatic immune and metabolic gene expression

**Figure 2:**
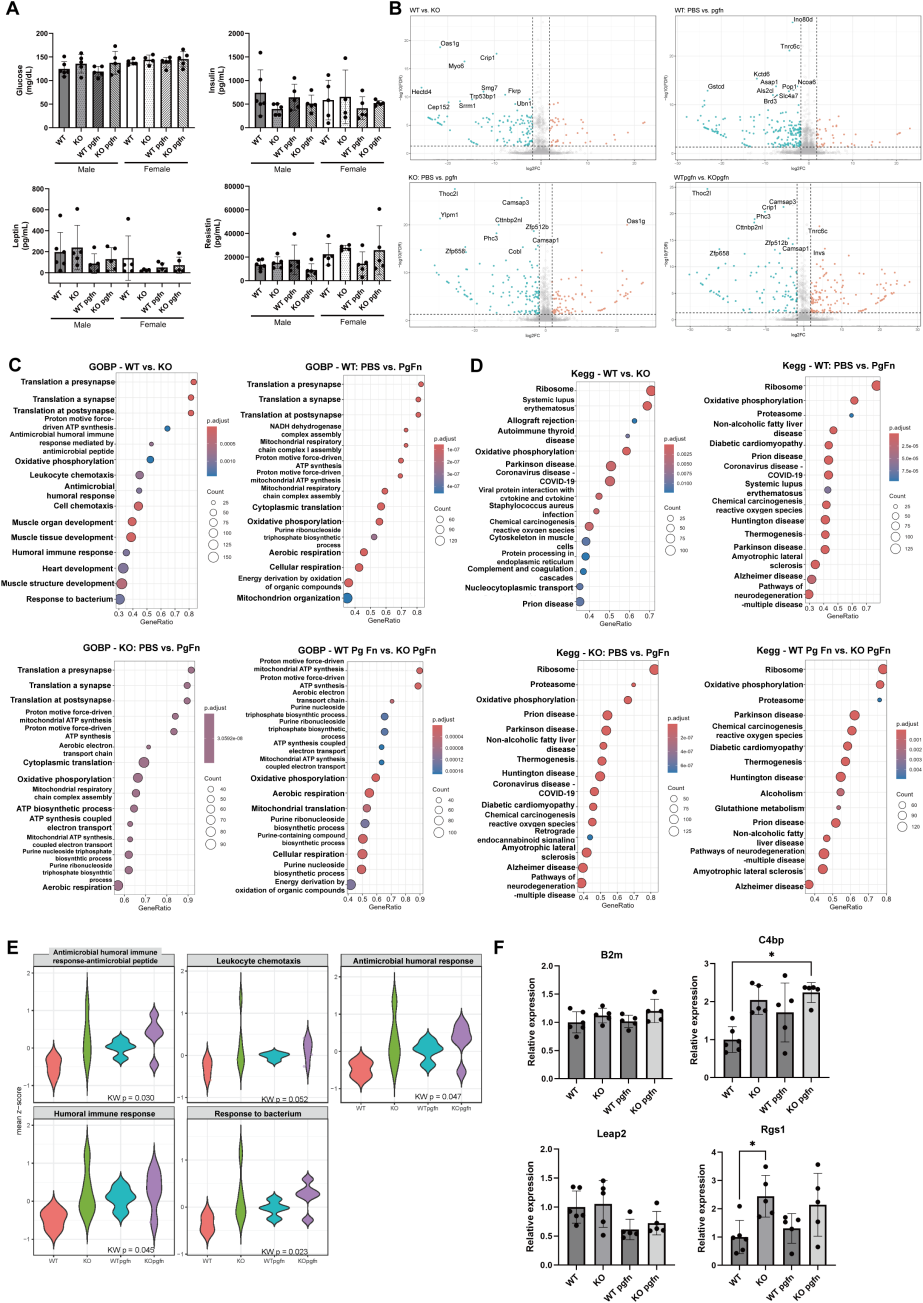
Metabolite and transcriptomic analysis of livers from WT and KO mice. **(A)** Serum levels of glucose, insulin, leptin, and resistin in WT and KO mice with or without periodontal pathogens gavage. Mean ± SD. **(B)** Differential gene expression analysis of liver transcriptomic sequencing data from WT and KO mice with or without pathogens gavage. **(C, D)** Gene Ontology biological process (GOBP) and Kyoto Encyclopedia of Genes and Genomes (KEGG) pathway enrichment analysis for each comparison, performed using gene set enrichment analysis (GSEA). **(E)** Mean Z-scores of genes from GOBP pathways significantly enriched in WT versus KO mice. **(F)** Relative expressions of inflammatory genes, *B2m, C4bp, Leap2, and Rgs1* mRNA in mice liver. Each dot represents an individual animal. Mean ± SD. Kruskal-Wallis test. *p< 0.05.

To evaluate the effect of CRIP1 deficiency on hepatic metabolic status, we measured serum levels of insulin, leptin, and resistin, as well as blood glucose. No significant differences were observed between WT and KO mice, and chronic exposure to periodontal pathogens similarly had no effect on these parameters (**Figure 2A**). Subsequently, RNA sequencing of liver tissues from both WT and CRIP1 KO mice, with or without periodontal pathogen gavage, revealed that CRIP1 deficiency and pathogen exposure each significantly altered hepatic gene expression profiles (**Figure 2B**). GO analysis comparing WT and KO mice identified enrichment of DEGs in pathways related to muscle development, antimicrobial, and immune responses, supporting the presence of an elevated basal pro-inflammatory state in KO mice that is consistent with hepatic *Crp* gene upregulation. KEGG analysis comparing WT and KO mice further revealed enrichment of DEGs in autoimmune and infection-related pathways (**Figures 2C, D**), suggesting that CRIP1 deficiency modulates hepatic immune regulatory networks even in the absence of overt inflammatory stimuli. However, analysis of individual genes exhibited a more complex pattern. Liver tissues from KO mice showed increased expression of both anti-inflammatory genes, such as *C4bp*, and pro-inflammatory genes, such as *Rgs1* (**Figures 2E, F**). This concomitant upregulation of genes with opposing inflammatory functions makes it difficult to define CRIP1.

### 3.3 CRIP1 is more likely to be associated with an anti-inflammatory role

**Figure 3:**
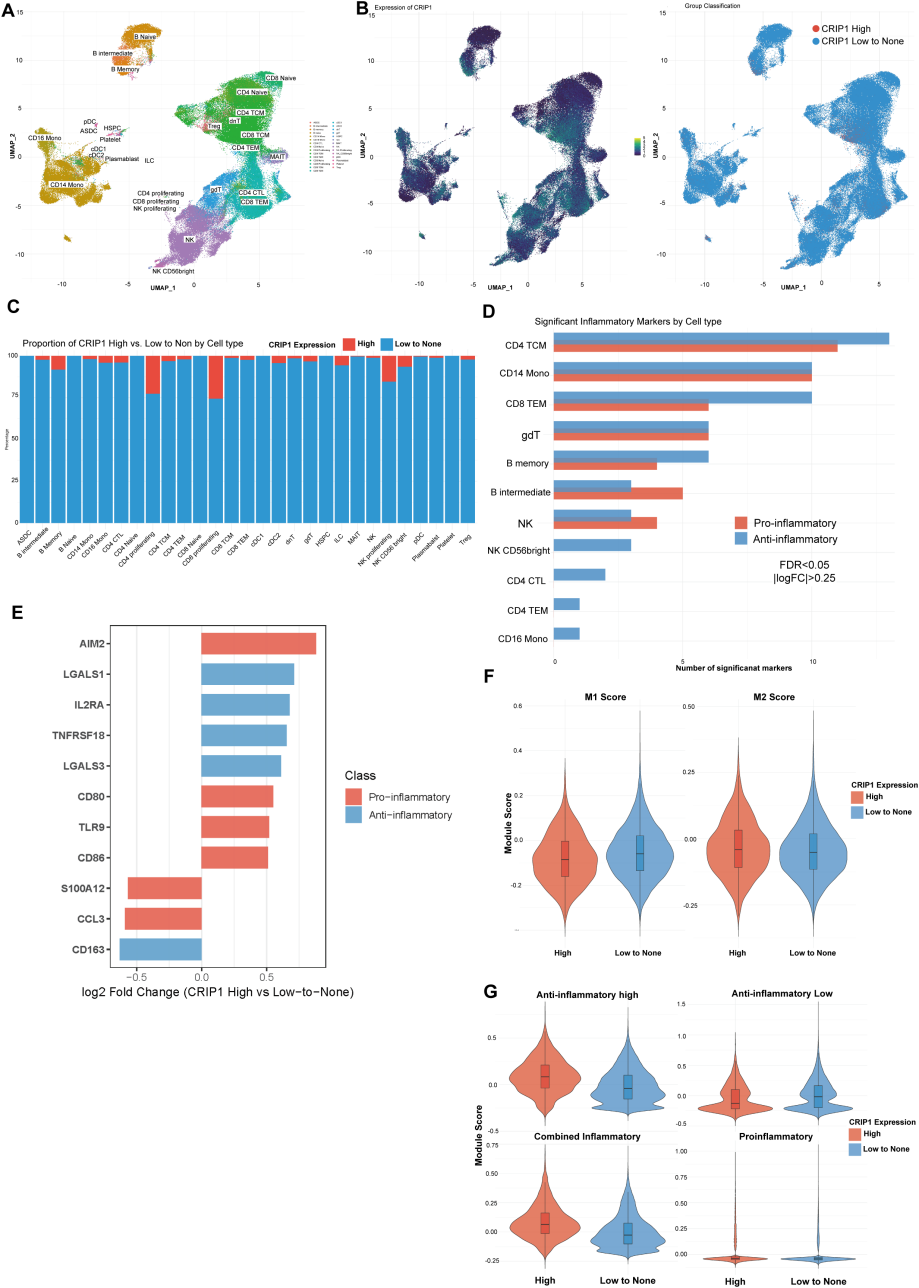
CRIP1 expression associates with inflammatory programs across peripheral immune cell subsets. **(A)** UMAP visualization of peripheral blood mononuclear cells (PBMC) of healthy and periodontitis patients, annotated into major immune cell populations. **(B)** UMAP plots showing CRIP1 expression (left) and classification of cells into CRIP1 High and CRIP1 Low-to-none groups (right). **(C)** Proportion of CRIP1 High versus Low-to-none cells within each immune cell subset. **(D)** Number of significantly upregulated pro-inflammatory and anti-inflammatory marker genes (FDR< 0.05, |log_2_FC| > 0.25) for each cell type when comparing CRIP1 High with Low-to-none cells. **(E)** Log_2_ fold change of selected differentially expressed inflammatory genes between CRIP1 High and Low-to-none groups, with genes classified as pro-inflammatory or anti-inflammatory. **(F)** Violin plots of M1-like and M2-like inflammatory module scores in CRIP1 High and Low-to-none cells. **(G)** Violin plots of module scores for anti-inflammatory high, anti-inflammatory low, combined inflammatory, and pro-inflammatory gene sets stratified by CRIP1 expression group. Boxplots indicate median and interquartile ranges.

Before clarifying the immunoregulatory role of CRIP1 using human data, we first examined the sequence homology between human and mouse species to address potential discrepancies between the two models and avoid misinterpretation of cross-species data integration. We found that human and mouse CRIP1 protein sequences are highly conserved, sharing 75 out of 77 amino acids (**Supplementary Figure 1**). This high degree of conservation suggests that analyses based on human data are appropriate. We therefore proceeded to investigate CRIP1 expression and function in human cells, analyzing genetic differences associated with CRIP1 expression using single-cell RNA sequencing data from our previous study on peripheral blood mononuclear cells from healthy individuals and periodontitis patients (**Figures 3A, B**). Quantitative analysis of the proportions of CRIP1-high and CRIP1-low expressing cells revealed a significantly higher frequency of CRIP1-high cells within the proliferating CD4+, proliferating CD8+, and proliferating NK cell populations, whereas other immune cell subsets exhibited markedly lower proportions of CRIP1-high expressing cells (**Figure 3C**). These findings suggest that CRIP1 is closely associated with activated and proliferating immune cells and may act as a regulator of immune cell activation. When inflammatory gene expression was compared across cell types, CRIP1 appeared to be linked to inflammation-suppressive programs. CD4+ T cell subsets predominantly expressed anti-inflammatory markers, whereas B intermediate and NK cells expressed pro-inflammatory markers (**Figure 3D**). To further define the role of CRIP1 in inflammation, we analyzed DEGs between CRIP1-high and CRIP1-low cells (log2FC > l0.5l, adjusted P value< 0.05) and found that expression levels of both pro-inflammatory and anti-inflammatory genes were differentially modulated according to CRIP1 status (**Figures 3E, F**). CRIP1-high cells showed a lower M1 and higher M2 ratio than CRIP1-low cells, and inflammatory module scoring demonstrated higher anti-inflammatory scores in CRIP1-high cells, supporting a likely anti-inflammatory role for CRIP1 in immune regulation (**Figure 3G**). To provide functional validation in immune cells, we overexpressed CRIP1 in THP-1 monocytes, which significantly suppressed the expression of pro-inflammatory cytokines, including IL-1β and IL-6 (**Supplementary Figure 2**). These findings support CRIP1’s anti-inflammatory function in immune cells.

### 3.4 CRIP1 deficiency affects zinc and iron metabolism-related genes in the liver

**Figure 4:**
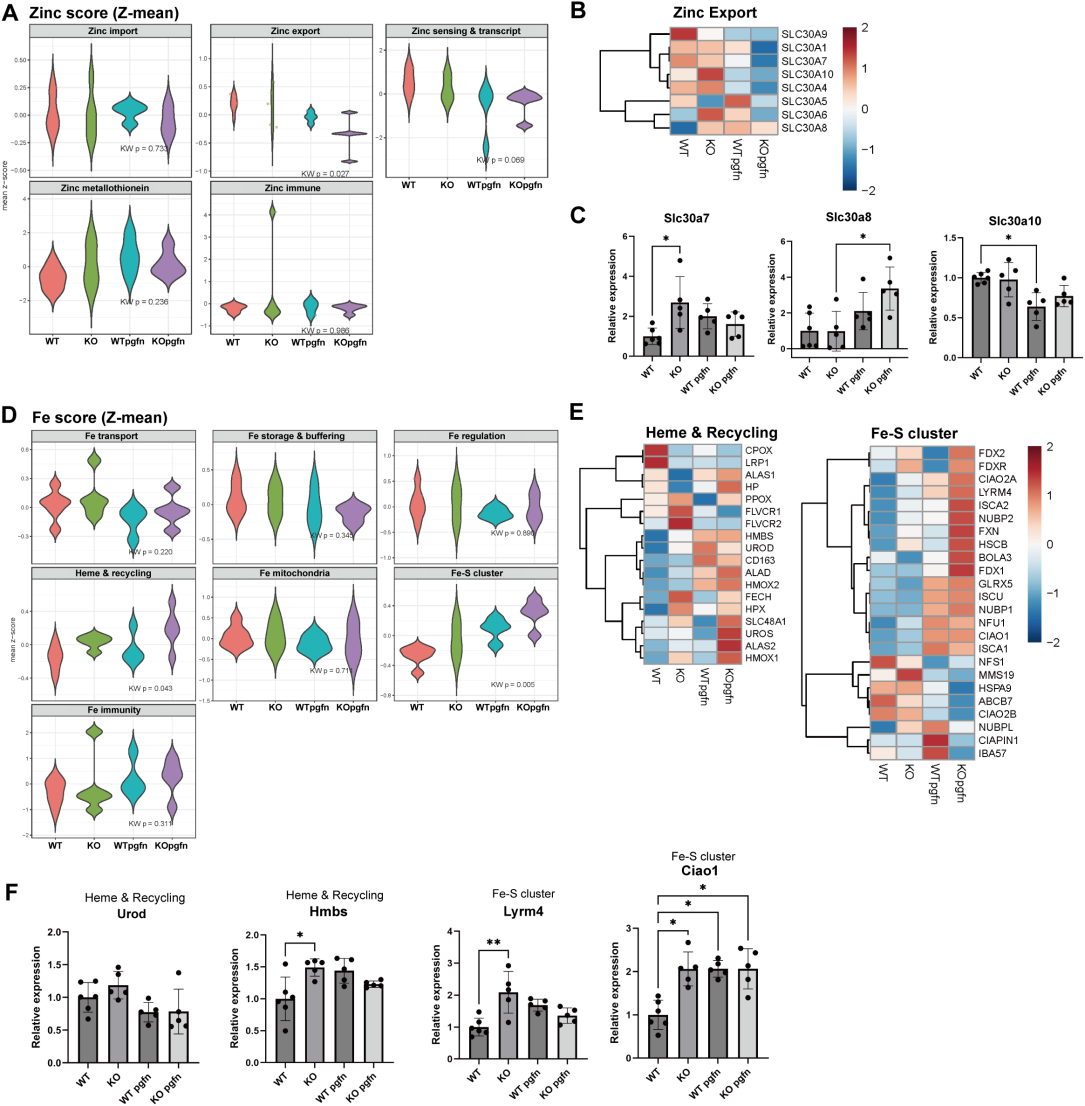
CRIP1 deficiency alters zinc and iron homeostasis. **(A)** Violin plots of zinc-related gene set scores (Z-mean) for zinc import, zinc export, zinc sensing and transcription, metallothioneins, and zinc-associated immune pathways in livers from WT and KO mice with or without pathogens gavage. **(B)** Heatmap of expression of zinc exporter genes (*Slc30a* family) across the four experimental groups. **(C)** Relative expression of *Slc30a7*, *Slc30a8*, and *Slc30a10* mRNA. **(D)** Violin plots of iron-related gene set mean Z-scores for iron transport, storage and buffering, regulation, heme synthesis and recycling, mitochondrial iron metabolism, Fe–S cluster biogenesis, and iron-related immune pathways. **(E)** Heatmaps of representative genes in the heme and recycling module and the Fe–S cluster module in liver tissue from each group. **(F)** Relative expression of *Urod* and *Hmbs* (heme and recycling) and *Lyrm4* and *Ciao1* (Fe–S cluster) mRNA. Each dot represents an individual animal. Mean ± SD. Kruskal-Wallist test. *p< 0.05.

CRIP1 is known to participate in zinc absorption and has been proposed to function as an intracellular zinc transport-related protein. Therefore, prior to analyzing the pathways affected by CRIP1 gene deficiency and pathogen exposure, we examined the impact of CRIP1 deficiency on genes associated with zinc transport activity. Among genes involved in zinc import, export, sensing, and interaction with metallothionein, an intracellular zinc reservoir, significant changes were observed in the zinc export family. In particular, expression of several *SLC30* family genes was increased in KO livers, suggesting compensatory upregulation of other zinc transporter genes in response to CRIP1 loss (**Figures 4A–C**). Considering the essential roles of iron in biological processes such as mitochondrial oxidative phosphorylation and its predominant metabolism in the liver, we next examined iron metabolism-related gene expression in WT and KO livers. Notably, among iron-metabolism-related genes, those involved in heme-iron recycling and iron-sulfur (Fe-S) cluster biogenesis were significantly altered in KO mice, as confirmed by qPCR (**Figures 4D–F**). These data suggest that CRIP1 may also affect iron metabolism, in addition to its involvement in zinc homeostasis, and may thereby contribute to broader metal ion-dependent metabolic regulation in the liver.

### 3.5 CRIP1 status and pathogen exposure modulate oxidative phosphorylation and insulin signaling pathways

**Figure 5:**
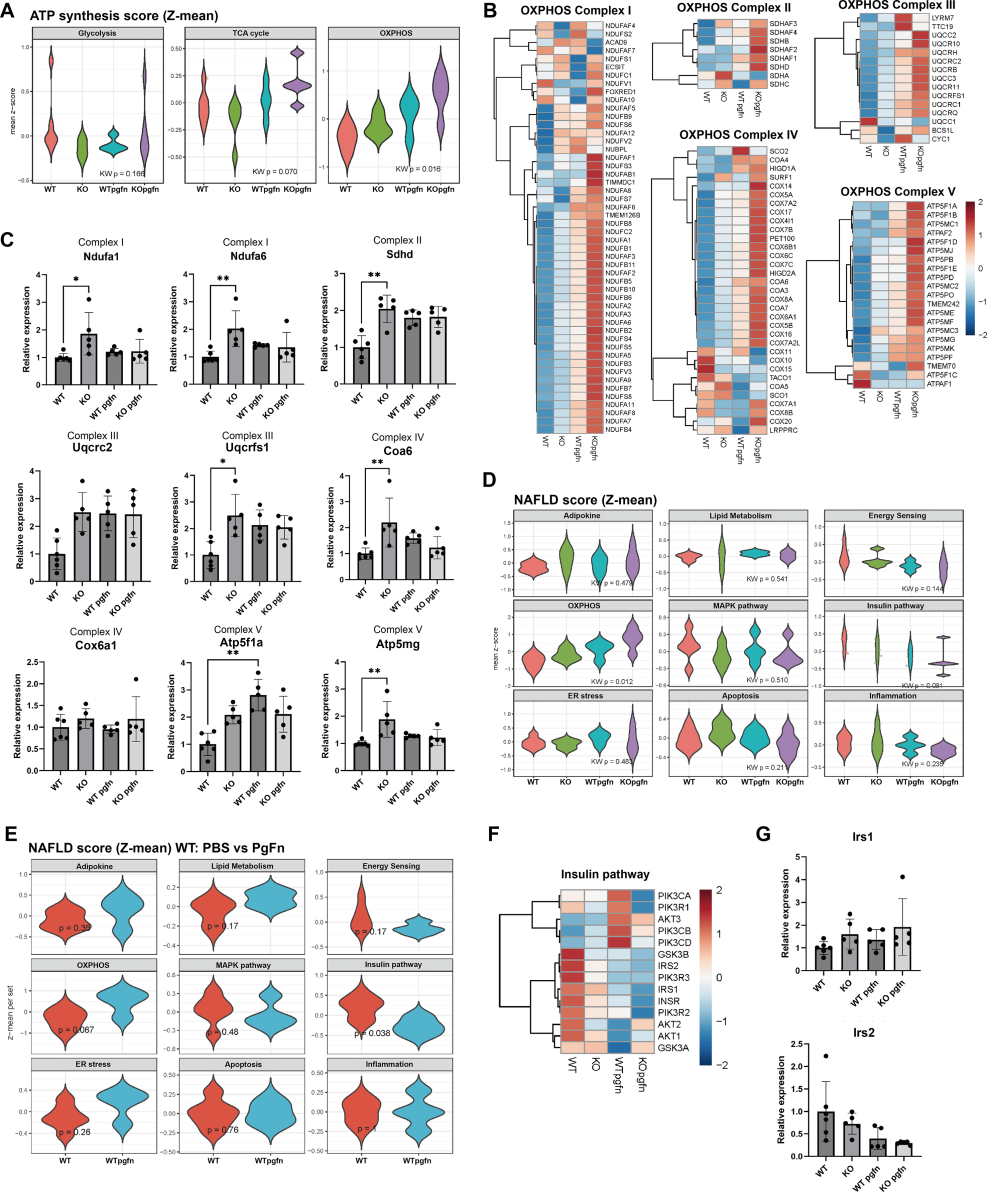
CRIP1 deficiency and periodontal pathogens modulate energy metabolism and insulin signaling. **(A)** Violin plots of ATP synthesis–related gene mean Z-scores for glycolysis, tricarboxylic acid (TCA) cycle, and oxidative phosphorylation (OXPHOS) across WT and KO mice with or without pathogens gavage. **(B)** Heatmaps of expression of genes encoding OXPHOS complexes I–V in the four experimental groups. **(C)** Relative expression of representative OXPHOS genes (*Ndufa1*, *Ndufa6*, *Sdhd*, *Uqcrc2*, *Coa6*, *Cox6a1*, *Atp5f1a*, and *Atp5mg*) mRNA. **(D)** Violin plots of nonalcoholic fatty liver disease (NAFLD)–related gene set scores (Z-mean) for adipokine signaling, lipid metabolism, energy sensing, OXPHOS, MAPK signaling, insulin pathway, endoplasmic reticulum (ER) stress, apoptosis, and inflammation in all four groups. **(E)** NAFLD-related gene set mean Z-scores in WT mice with PBS versus periodontal pathogen gavage, shown as paired violin plots for each pathway. **(F)** Heatmap of expression of genes in the insulin signaling pathway. **(G)** Relative expression of *Irs1* and *Irs2* mRNA in each group. Each dot represents an individual animal. Mean ± SD. Kruskal-Wallis test. *p< 0.05. **p<0.01.

We analyzed DEGs involved in energy production across the primary stages of ATP synthesis: glycolysis, the Krebs cycle, and oxidative phosphorylation. Among these pathways, only genes related to oxidative phosphorylation were significantly and consistently altered (**Figure 5A**). We therefore examined fold changes in genes encoding components of the five oxidative phosphorylation complexes (OXPHOS). Although the initial analysis suggested that pathogen-exposed KO mice exhibited the most prominent alterations, validation revealed that the absence of CRIP1 per se had the primary impact on OXPHOS gene expression. Pathogen exposure induced modest increases in gene expression, but these changes were generally less pronounced than those observed in KO mice or were statistically insignificant. Most genes showed similar or lesser degrees of upregulation in both KO and WT mice following pathogen exposure, implying that CRIP1 status is a more decisive factor in the regulation of oxidative phosphorylation than pathogen exposure (**Figures 5B, C**). To complement the transcriptomic metabolic profiling, we performed ATP quantification assays in HepG2 cell with CRIP1 overexpression. Consistent with our transcriptomics findings, CRIP1 overexpression significantly reduced intracellular ATP production in hepatocytes, whereas periodontal pathogen exposure had no significant effect (**Supplementary Figure 3**). These findings further emphasize a role for CRIP1 in regulating cellular energy metabolism.

Additionally, periodontal pathogen exposure was associated with expression changes related to non-alcoholic liver disease (NAFLD), suggesting periodontitis as a potential contributor to NAFLD progression. KEGG analysis (**Figure 2D**) indicated enrichment of NAFLD-related pathways in pathogen-exposed mice. Accordingly, we analyzed NAFLD-associated genes and observed significant changes in genes related to oxidative phosphorylation (**Figure 5D**). Moreover, genes associated with insulin signaling exhibited decreased expression, although statistical significance was marginal (p=0.081). Given previous reports of a strong correlation between chronic infection and NAFLD (14, 15), we further examined NAFLD-related gene expression specifically in WT mice depending on pathogen exposure. This analysis revealed that pathogen exposure markedly downregulated insulin signaling pathways, and validation confirmed significant suppression of the *Irs2* expression (**Figures 5E–G**). Although transcriptomic changes associated with NAFLD pathways were not prominent in CRIP1 KO mice without periodontal pathogen exposure, liver histology (N = 5) revealed increased lobular inflammation in both CRIP1 KO mice and pathogen-gavaged WT mice compared to WT controls. These findings further support CRIP1’s anti-inflammatory role *in vivo* (**Supplementary Figure 4**). These findings suggest a potential early mechanistic link between chronic low-grade inflammation, such as periodontitis, and hepatic insulin signaling impairment, which may contribute to the initiation of insulin resistance and NAFLD.

## 4 Discussion

Numerous knockout mouse studies have demonstrated that loss of gene function often leads to significant alterations in body weight and growth, with up to 35% of viable KO strains exhibiting lower body weight than WT controls (16). In addition, *Znt7* KO mice, which are deficient in one of the zinc transporters, displayed limited body weight gain and impaired growth (17). In contrast, the observation of normal growth and no significant difference in body weight gain between CRIP1 KO and WT mice in this study suggests that CRIP1 is not a critical factor for growth and development, despite its established role in zinc homeostasis. Alternatively, this finding may reflect compensatory upregulation of other genes within the zinc transporter network. Previous studies have shown that other zinc transporter family members such as *SLC30* (*ZnT*) and *SLC39* (*ZIP*) can be upregulated in response to zinc deficiency or loss of a specific zinc transporter (18, 19), a phenomenon also observed in our study, thereby preserving normal growth trajectories in CRIP1-deficient mice. Taken together, these data indicate that either CRIP1’s function is dispensable for growth, or robust compensatory mechanisms operate in its absence, underscoring the resilience of systemic zinc regulation *in vivo (*17).

Although CRIP1 was initially noted for its role in zinc transport, early studies also suggested a potential function as an immune regulatory protein. However, whether this role is pro-inflammatory or anti-inflammatory has remained unclear (8). To date, few studies have directly addressed the relationship between CRIP1 and inflammation, and existing findings have been inconclusive. Notably, Lorraine Lanningham-Foster et al. reported that transgenic mice overexpressing CRIP1 exhibited increased susceptibility to LPS and viral infection, manifested by higher lethality and greater weight loss (20). This would seem to support a pro-inflammatory role for CRIP1. However, a cytokine analysis in these mice revealed decreased serum interferon-γ (IFN-γ) and increased levels of both IL-6 and IL-10 in response to LPS and viral exposure. This cytokine shift suggests dysregulated or compensatory immune modulation rather than straightforward immune activation. Our own experiments demonstrated that CRIP1-deficient mice were relatively unresponsive to pathogen challenge, with no significant changes in IL-6 or TNF-α levels compared to controls. This lack of response may suggest impaired pro-inflammatory signaling, but could also reflect a reduction in overall immune reactivity, possibly due to a regulatory or suppressive function of CRIP1 under inflammatory conditions. Furthermore, single-cell analysis of PBMC from patients with chronic inflammatory status suggested that CRIP1 is an immune regulatory factor that is closer to an anti-inflammatory role, although finely modulated by the surrounding immune network. Taken together, these findings underscore the complex and context-dependent nature of CRIP1’s role in immune regulation and highlight the need for further mechanistic studies to determine whether CRIP1 primarily promotes or restrains inflammatory responses.

In addition to its immune regulatory function, our findings indicate that CRIP1 may participate in broader aspects of cellular metabolism, including zinc transport, iron metabolism, and oxidative phosphorylation. Although CRIP1 has long been presumed to function as a zinc transporter, definitive mechanistic evidence supporting this role or its relationship with other zinc transporters, remains inconclusive. A recent study in gastric cancer suggested that CRIP1 may act as a non-classical zinc transporter involved in the regulation of lymphoangioenesis, but this observation lacked molecular validation (21). In our analysis, CRIP1 expression showed a significant positive correlation with genes of the *SLC30* family, which encode classical zinc exporters, supporting its potential involvement in zinc homeostasis. In addition, our data revealed associations between CRIP1 and genes implicated in iron metabolism, particularly those related to Fe-S cluster biogenesis and heme recycling. Birkenmeier EH et al. previously proposed a possible link between CRIP1 and iron metabolism, but this hypothesis was largely speculative, based on protein sequence similarity, and has required direct experimental validation (1). Because iron plays essential roles in cellular redox regulation, Fe-S cluster assembly, apoptosis, and autophagy (22–24), our findings may provide mechanistic insight into prior observations connecting CRIP1 to cellular homeostasis and stress responses. Our results also imply a potential relationship between CRIP1 and oxidative phosphorylation. This finding is consistent with previous bioinformatics analyses showing a positive association between CRIP1 expression and oxidative phosphorylation pathways, including GO enrichment and KEGG-based GSEA in varicose vein tissues (25) as well as experimental evidence in melanoma cells showing altered expression of OXPHOS-related proteins following CRIP1 modulation (26). Although these observations collectively suggest a possible connection between CRIP1 and mitochondrial function, it remains to be clarified whether CRIP1 directly influences mitochondrial energy metabolism or acts indirectly through zinc- and iron-related processes. Taken together, our findings support the hypothesis that CRIP1 may function as a mediator linking metal ion metabolism with mitochondrial oxidative pathways. Further mechanistic and functional studies are warranted to substantiate this hypothesis and elucidate how CRIP1 contributes to metabolic and inflammatory regulation in the context of disease progression.

## Data Availability

The datasets presented in this study can be found in online repositories. The names of the repository/repositories and accession number(s) can be found below: PRJNA1377028 (Bioproject, NCBI).
