# Supplementary material for: Molecular insights into CRIP1 as an immunometabolic regulator revealed by CRIP1 knockout and single-cell transcriptomics
Source: Front Immunol. 2026 Mar 26;17:1762474. doi: 10.3389/fimmu.2026.1762474 (PMC13061677; doi:10.3389/fimmu.2026.1762474)
Supplement: Supplementary file 1 [file Table1.docx]

**Supplementary Table 1.**

| **Gene Names (Mouse)** | **Primer Sequence** | **Length** |
| --- | --- | --- |
| *Atp5f1a*_F | GTG ATG GTA TTG CGC GAG TG | 20 |
| *Atp5f1a*_R | CGG GTT CCA AGT TCA GGG AC | 20 |
| *Atp5mg*_F | AAC CCC TGC TGA AAT CCC TAC | 21 |
| *Atp5mg*_R | AAA CCA CAT CCA CAC CTC AGT | 21 |
| *B2m*_F | CTC GGT GAC CCT GGT CTT TC | 20 |
| *B2m*_R | GGA TTT CAA TGT GAG GCG GG | 20 |
| *C4bp*_F | CTG CAC ATG GGA GGC TTC ATA | 21 |
| *C4bp*_R | AGG GCA TTG GGT ATA GCA GG | 20 |
| *Ciao1*_F | GCT CTC ATA CGC AGG ACG TT | 20 |
| *Ciao1*_R | CAC AGC AGA CCC AGT CAT CC | 20 |
| *Coa6*_F | TGG ACG ACA ACG CGG A | 16 |
| *Coa6*_R | AAT CCT CCT GCT TCA AAT TTT TCC T | 25 |
| *Cox6a1*_F | CTC AAC GTG TTC CTC AAG TCG | 21 |
| *Cox6a1*_R | GGT TGT GGA AGA GGG TAT GGT | 21 |
| *Crp*_F | GAA GCT ACT CTG GTG CCT TCT | 21 |
| *Crp*_R | AGT GGC TTC TTT GAC TCT GCT T | 22 |
| *Gapdh*_F | CAC TTG AAG GGT GGA GCC AAA | 21 |
| *Gapdh*_R | GAC TGT GGT CAT GAG CCC TT | 20 |
| *Hmbs*_F | TTC GGG GAA ACC TCA ACA CC | 20 |
| *Hmbs*_R | CTC CCT GAC CCA CAG CAT AC | 20 |
| *Irs1*_F | GCT GGG GGT TTG GAG AAG AG | 20 |
| *Irs1*_R | TTG GGG AGT TGC CCT CAT TG | 20 |
| *Irs2*_F | CAC AAC CTA TCG TGG CAC CT | 20 |
| *Irs2*_R | AAG GTC TCT GAA CTG TGG CG | 20 |
| *Leap2*_F | CTC TTT GCA GTG CTC CTG ACT | 21 |
| *Leap2*_R | CCA ATG GGC CTG AGG GAA AC | 20 |
| *Lyrm4*_F | TGT ACC GGG CGA TGA TGA GA | 20 |
| *Lyrm4*_R | TGG ACC TGT CTG CGG ATT ATT | 21 |
| *Ndufa1*_F | AAA TTC ACC AAC GGG GGC A | 19 |
| *Ndufa1*_R | CGT CTA TCG CGT TCC ATC AG | 20 |
| *Ndufa6*_F | GTA CCT CGG TGA AGC CCA TT | 20 |
| *Ndufa6*_R | ATC CCG TCC TTG TTT CAC CG | 20 |
| *Rgs1*_F | AAA CTC CTT GCC AAC CAG ACA | 21 |
| *Rgs1*_R | ATA GTC CTC ACA AGC CAA CCA | 21 |
| *Sdhd*_F | GAA TCC CTG CTC TGT GGT GG | 20 |
| *Sdhd*_R | GGT GTC CCC ATG AAC GTA GT | 20 |
| *Slc30a7*_F | TGG TGT AAT TGC CTC TGC CAT | 21 |
| *Slc30a7*_R | ACG AGG GAG GAG TTC TTT GC | 20 |
| *Slc30a8*_F | TGG CTA TCC TCA CTG ATG CG | 20 |
| *Slc30a8*_R | CAG TCA CCA CCC AGA TGC AA | 20 |
| *Slc30a10*_F | CAA GGG GGT CAA CAT GGA AGA | 21 |
| *Slc30a10*_R | CGT GCC CTT CTG GTG TTT GA | 20 |
| *Uqcrc2*_F | ATG CTC CTC TGT CAA GAA TCG G | 22 |
| *Uqcrc2*_R | TGC TTC AAT CCC ACG GGT TAT | 21 |
| *Uqcrfs1*_F | TCT GGA TGT GAA GCG ACC CT | 20 |
| *Uqcrfs1*_R | CGA TAG TCA GAG AAG TCG GGC | 21 |
| *Urod*_F | GCC CTA CAT TCG TGA TGT GGC | 21 |
| *Urod*_R | CAC TGC CTT CCC GAC ACG TT | 20 |

**Supplementary Table 2.**

| **Gene Names (Human)** | **Primer Sequence** | **Length** |
| --- | --- | --- |
| *ATP5F1A*_F | CCC AGT TCG GTT CTG ACC TC | 20 |
| *ATP5F1A* _R | TCC CCT TAC ACC CGC ATA GA | 20 |
| *C4BPA*_F | TGG CTA TGG TGT GGT TGG TC | 20 |
| *C4BPA*_R | CCT CTG GGT TTG GGA GAC AC | 20 |
| *CIAO1*_F | GCT CTG ACC CCA GTT GGA AA | 20 |
| *CIAO1*_R | TTG ACA TCC TGG GAA TGG GC | 20 |
| *COA6*_F | CGG GAT GAG TAC TGG AAG TGT | 21 |
| *COA6*_R | GAA GGC TCA AAT TGT CCT GCT TC | 23 |
| *CRP*_F | CTT GAC CAG CCT CTC TCA TGC | 21 |
| *CRP*_R | AAC GGT GCT TTG AGG GAT ACA | 21 |
| *GAPDH*_F | CAA ATT CCA TGG CAC CGT CAA | 21 |
| *GAPDH*_R | AGC ATC GCC CCA CTT GAT TT | 20 |
| *HMBS*_F | GCC AGA GAA GAG TGT GGT GG | 20 |
| *HMBS*_R | CTG AAC TCC TGC TGC TCG TC | 20 |
| *IRS2*_F | CCT GCC CCC TGC CAA CAC CT | 20 |
| *IRS2*_R | TGT GAC ATC CTG GTG ATA AAG CC | 23 |
| *NDUFA6*_F | CAG TCG GGA CAT GAA CGA GG | 20 |
| *NDUFA6*_R | TGT GTC CGC TGC TTC CAT AC | 20 |
| *RNA18S*_F | GGC CGT TCT TAG TTG GTG GA | 20 |
| *RNA18S*_R | CCC GGA CAT CTA AGG GCA TC | 20 |
| *SDHD*_F | CGG TTC TCT GGA GGC TGA G | 19 |
| *SDHD*_R | TGG GAT AGG TCG GTC CTG AA | 20 |
| *SLC30A7*_F | AGT CTG CCT CAG TGC TAT CAG | 21 |
| *SLC30A7*_R | TCC ACC TAG CAT CAG CAT CA | 20 |
| *UQCRFS1*_F | CCT TTG GTC GCC TCC GT | 17 |
| *UQCRFS1*_R | AAA CTT CAA GGC GGC GGT ATT | 21 |

**Supplementary Table 3.**

| **M1 macrophage markers** | \| *PTGS2* \| *FCGR1A* \| *IL12A* \| *NFKB1* \| \| --- \| --- \| --- \| --- \| \| *CD80* \| *FCGR2A* \| *IL23A* \| *RELA* \| \| *CD86* \| *SLAMF7* \| *CXCL10* \| *HIF1A* \| \| *CD40* \| *TLR2* \| *CCL5* \| *PFKFB3* \| \| *IL1B* \| *TLR4* \| *CCL2* \| *PKM* \| \| *IL6* \| *STAT1* \| *CD38* \| *IDO1* \| \| *TNF* \| *IRF5* \|  \|  \| | | |
| --- | --- | --- | --- | --- | --- | --- | --- | --- | --- | --- | --- | --- | --- | --- | --- | --- | --- | --- | --- | --- | --- | --- | --- | --- | --- | --- | --- | --- | --- | --- | --- |
| **M2 macrophage markers** | \| *MRC1* \| *TGFB1* \| *FOLR2* \| *FN1* \| \| --- \| --- \| --- \| --- \| \| *CD163* \| *IL1RN* \| *STAT6* \| *TIMP1* \| \| *MSR1* \| *IL4R* \| *PPARG* \| *PDGFA* \| \| *CLEC10A* \| *IL13RA1* \| *IRF4* \| *PDGFB* \| \| *STAB1* \| *CD200R1* \| *KLF4* \| *PPARGC1B* \| \| *IL10* \| *FCGR2B* \| *VEGFA* \| *CPT1A* \| \| *MARCO* \|  \|  \| | | |
| **Proinflammatory markers** | Pro-inflammatory cytokines | *IL1B*  *IL6*  *TNF*  *IL12A*  *IL12B*  *IL18*  *IL23A* | *IFNG*  *IL17A*  *IL17F*  *IL15*  *IL21*  *LTA*  *LTB* |
|  | Pro-inflammatory chemokines | *CCL2*  *CCL3*  *CCL4*  *CCL5*  *CCL20*  *CXCL1*  *CXCL2* | *CXCL8*  *CXCL9*  *CXCL10*  *CXCL11*  *CCL7*  *CCL8*  *CCL11*  *CCL13* |
|  | Inflammasome | *NLRP3*  *NLRP1*  *NLRC4*  *AIM2* | *PYCARD*  *CASP1*  *CASP4*  *CASP5* |
|  | Pattern recognition receptors | *TLR1*  *TLR2*  *TLR4*  *TLR6*  *TLR7* | *TLR8*  *TLR9*  *NOD1*  *NOD2* |
|  | Pro-inflammatory transcription factors | *NFKB1*  *NFKB2*  *RELA*  *RELB*  *JUN*  *FOS* | *STAT1*  *STAT3*  *STAT4*  *IRF1*  *IRF5*  *IRF7* |
|  | Cell adhesion and activation | *ICAM1*  *VCAM1*  *SELE*  *SELP*  *CD69* | *CD25*  *PTGS2*  *NOS2*  *CD40*  *CD80*  *CD86* |
|  | Matrix metalloproteinases | *MMP1*  *MMP2*  *MMP3*  *MMP7* | *MMP8*  *MMP9*  *MMP12*  *MMP13* |
|  | Reactive oxygen species | *CYBB*  *NCF1*  *NCF2*  *NCF4* | *CYBA*  *NOX1*  *NOX4* |
|  | S100 alarmins (pro-inflammatory) | *S100A8*  *S100A9* | *S100A12*  *HMGB1* |
| **Anti-inflammatory markers** | Anti-inflammatory cytokines | *IL10*  *IL4*  *IL13*  *TGFB1*  *TGFB2*  *TGFB3* | *IL1RN*  *IL11*  *IL19*  *IL35*  *IL37*  *IL38* |
|  | Regulatory chemokines | *CCL17*  *CCL22* | *CCL24*  *CXCL12* |
|  | Regulatory T cell markers | *FOXP3*  *IL2RA*  *CTLA4*  *TNFRSF18* | *IKZF2*  *LGALS1*  *LGALS3*  *LGALS9* |
|  | Immune checkpoints (inhibitory) | *PDCD1*  *CD274*  *PDCD1LG2*  *LAG3* | *TIGIT*  *HAVCR2*  *BTLA*  *CD200*  *CD200R1* |
|  | SOCS proteins (negative regulators) | *SOCS1*  *SOCS2* | *SOCS3*  *CISH* |
|  | Anti-inflammatory transcription factors | *PPARG*  *PPARD*  *NR3C1*  *NFKBIA*  *NFKBIB* | *NFKBIE*  *DUSP1*  *DUSP2*  *STAT6*  *IRF4* |
|  | Lipid mediators and enzymes | *ALOX15*  *ALOX5*  *PTGES*  *PTGS1* | *HPGD*  *LTA4H*  *PTGDS* |
|  | M2 macrophage markers | *CD163*  *MRC1*  *MSR1*  *ARG1*  *CHIL3* | *RETNLA*  *CCL18*  *CLEC10A*  *STAB1* |
|  | Tissue repair and remodeling | *AREG*  *HGF*  *VEGFA*  *VEGFB*  *PDGFA* | *PDGFB*  *FGF2*  *EGF*  *IGF1*  *COL1A1* |
|  | Other anti-inflammatory molecules | *ANXA1*  *HMOX1*  *NQO1* | *PTPN22*  *ENTPD1*  *NT5E* |
