# Supplementary material for: Molecular insights into CRIP1 as an immunometabolic regulator revealed by CRIP1 knockout and single-cell transcriptomics
Source: Front Immunol. 2026 Mar 26;17:1762474. doi: 10.3389/fimmu.2026.1762474 (PMC13061677; doi:10.3389/fimmu.2026.1762474)
Supplement: Supplementary file 2 [file Table2.docx]

**Supplementary Materials and Methods**

**Cell culture and treatments**

HepG2 cells were purchased from the Korean Cell Line Bank (Korean Cell Line Research Foundation, Seoul, Korea), and THP-1 cells were obtained from the American Type Culture Collection (ATCC, Manassas, VA, USA). HepG2 and THP-1 cells were cultured in Minimum Essential Medium (MEM) and Roswell Park Memorial Institute 1640 medium (RPMI-1640), respectively (Welgene, Daegu, Korea). The CRIP1 overexpression vector was transfected using Lipofectamine™ 3000 reagent (Invitrogen, MA, USA). For THP-1 cells, phorbol 12-myristate 13-acetate (PMA; MedChemExpress, NJ, USA) was applied at a concentration of 250 ng/mL for 24 h to induce differentiation, followed by replacement with fresh medium prior to transfection.

**Adenosine triphosphate (ATP) quantification assay**

Cellular ATP levels in HepG2 cells were measured using a colorimetric ATP Assay Kit (#ab83355, Abcam, Cambridge, UK) according to the manufacturer’s instructions. Briefly, 1.0 × 10^6 cells were seeded and subjected to the indicated treatments. Cells were harvested using 0.05% trypsin/EDTA and washed with ice-cold phosphate-buffered saline (PBS). The cells were resuspended in ATP assay buffer and centrifuged at 13,000 × g for 5 min at 4°C. The supernatants were collected, and 50 μL of each sample or standard was loaded into a 96-well plate. The ATP reaction mix was then added, and after 30 min of incubation, absorbance was measured at 570 nm using a microplate reader (BioTek Synergy HT, Agilent, CA, USA).

**Formalin-fixed paraffin-embedded (FFPE) tissue preparation and hematoxylin and eosin (H&E) staining**

Mouse livers were fixed in 4% paraformaldehyde, followed by dehydration through a graded ethanol series, clearing in xylene, and embedding in paraffin. Paraffin-embedded liver tissues were sectioned using a microtome (Leica Biosystems, Deer Park, IL, USA) and mounted on glass slides.
For H&E staining, mounted sections were dried, deparaffinized in xylene, and rehydrated through a graded ethanol series to water. The sections were then stained with hematoxylin, rinsed in distilled water, and counterstained with eosin. After staining, the sections were dehydrated through graded ethanol and coverslipped using a mounting medium.
