# Supplementary material for: Molecular insights into CRIP1 as an immunometabolic regulator revealed by CRIP1 knockout and single-cell transcriptomics
Source: Front Immunol. 2026 Mar 26;17:1762474. doi: 10.3389/fimmu.2026.1762474 (PMC13061677; doi:10.3389/fimmu.2026.1762474)
Supplement: Supplementary file 3 [file Table3.docx]

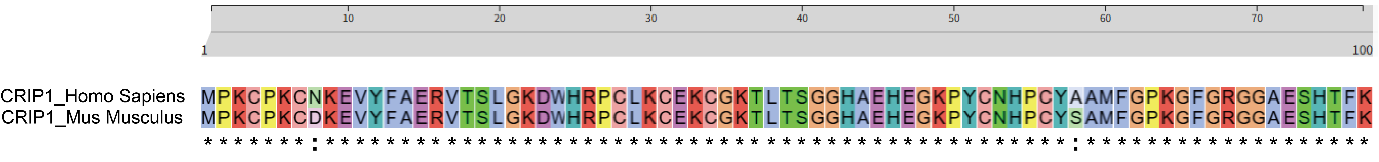


**Supplementary Fig. 1. Sequence homology of CRIP1 protein in *Homo Sapiens* and *Mus Musculus***

Human and mouse CRIP1 protein sequences are highly conserved, sharing 97.4% amino acid identity, with 2 amino acid differences out of 77 residues.
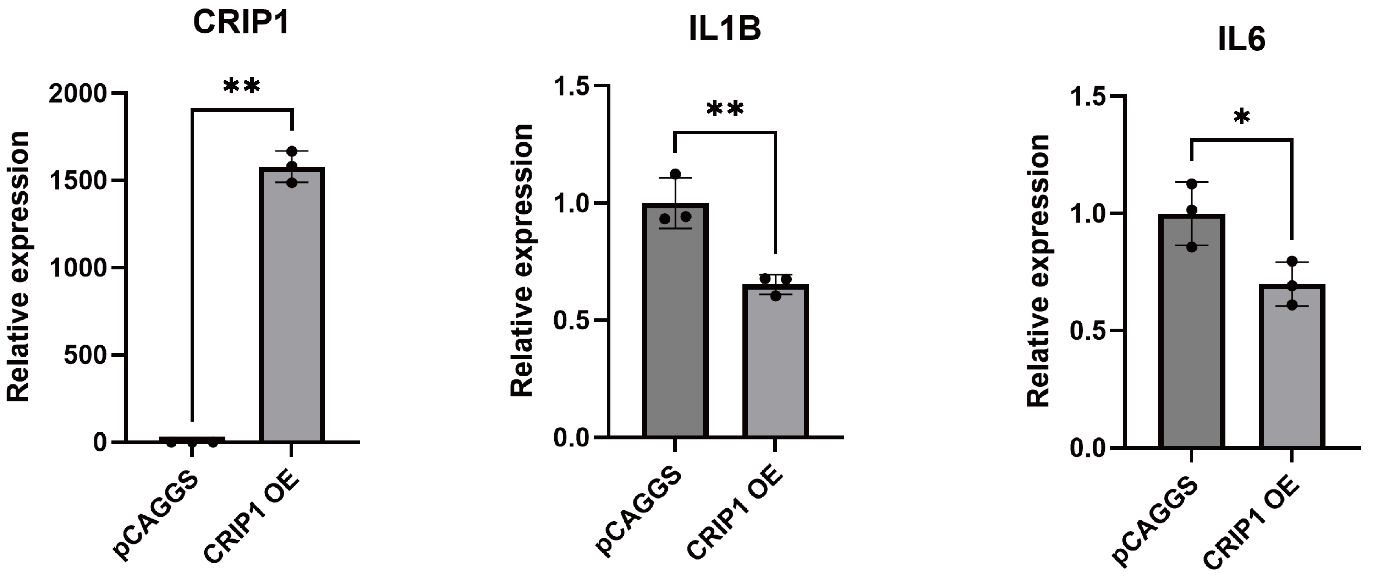


**Supplementary Fig. 2. Inhibition of pro-inflammatory cyotkines by CRIP1 ectopic expression in THP-1.**

Relative expression of pro-inflammatory cytokines *IL1B* and *IL6* in THP-1 cells with or without CRIP1 overexpression.

Mean ± SD. Mann-Whitney u test. *p < 0.05. **p<0.01. CRIP1 OE: CRIP1 overexpression.


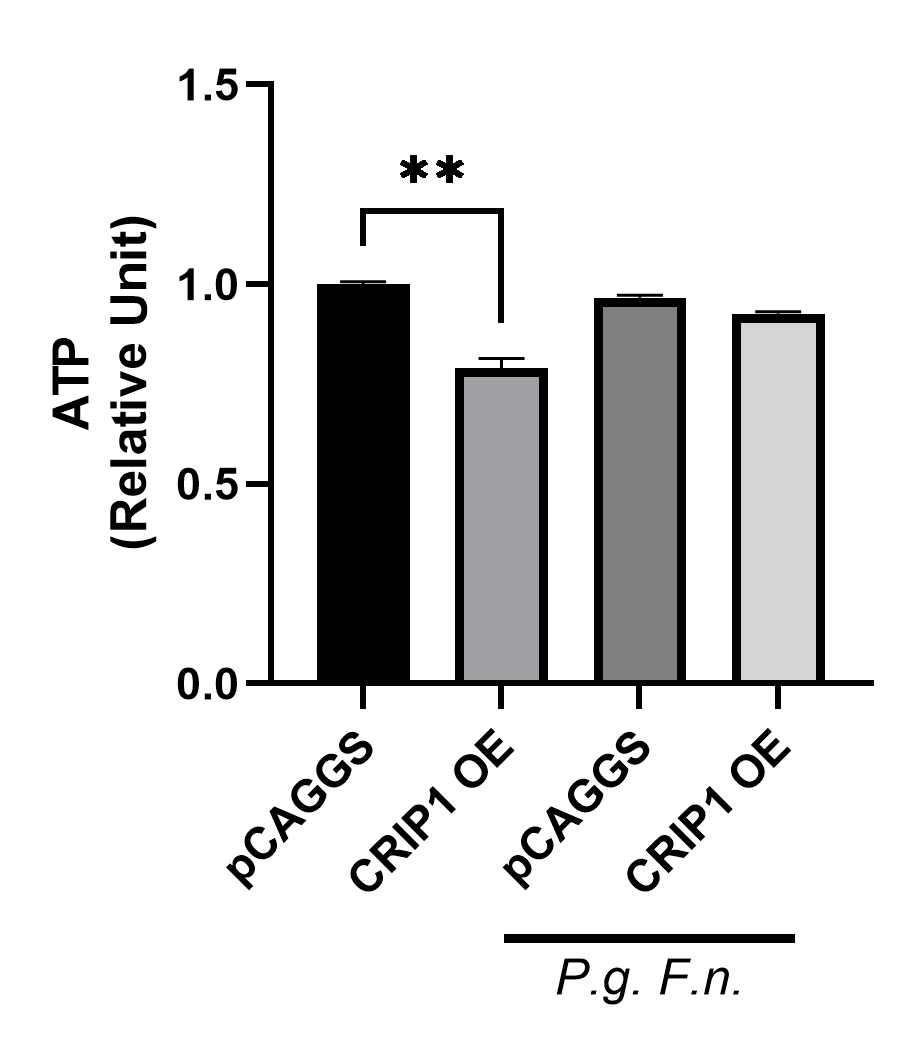


**Supplementary Fig. 3. Intracellular adenosine triphosphate (ATP) amounts were decreased in CRIP1 overexpressed HepG2.**

Normalized relative ATP levels in HepG2 cells following CRIP1 overexpression and stimulation with *P. gingivalis*, *F. nucleatum*.

Mean ± SD. Kruskal-Wallis test. *p < 0.05. P.g.: *P. gingivalis,* F.n*. : F. nucleatum.* CRIP1 OE: CRIP1 overexpression.


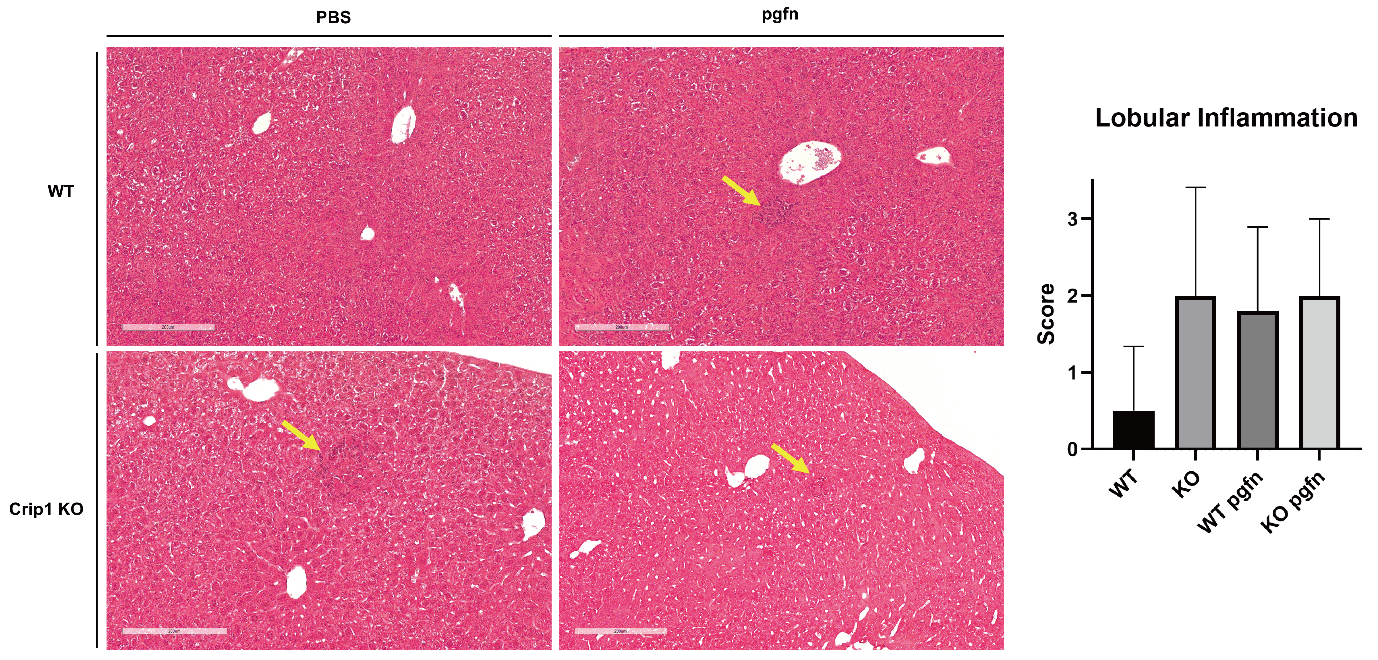


**Supplementary Fig. 4. Histological change of liver of oral gavaged mice and insulin signaling pathway alteration by periodontal pathogen.**

Representative images of hematoxylin and eosin (H&E)-stained mouse liver sections. Yellow arrows indicate immune cell infiltration foci. The bar graph shows lobular inflammation scores. Mean ± SD.

Pgfn: *P. ginigivalis* and *F. nucleatum.*
